# DDX3 DEAD-box RNA helicase (*Hel67*) gene disruption impairs infectivity of *Leishmania donovani* and induces protective immunity against visceral leishmaniasis

**DOI:** 10.1038/s41598-020-75420-y

**Published:** 2020-10-26

**Authors:** Satish Chandra Pandey, Veena Pande, Mukesh Samant

**Affiliations:** 1 Cell and Molecular Biology Laboratory, Department of Zoology, Kumaun University, SSJ Campus, Almora, Uttarakhand India; 2 Department of Biotechnology, Kumaun University, Bhimtal Campus, Nainital, Uttarakhand India

**Keywords:** Biotechnology, Immunology

## Abstract

Visceral leishmaniasis (VL) is a vector-borne disease caused by the digenetic protozoan parasite *Leishmania donovani* complex. So far there is no effective vaccine available against VL. The DDX3 DEAD-box RNA Helicase (*Hel67*) is 67 kDa protein which is quite essential for RNA metabolism, amastigote differentiation, and infectivity in *L. major and L. infantum*. To investigate the role of *Hel67* in the *L. donovani*, we created *L. donovani* deficient in the *Hel67*. Helicase67 null mutants (*LdHel67*^*−/−*^) were not able to differentiate as axenic amastigotes and were unable to infect the hamster. So, we have analyzed the prophylactic efficacy of the *LdHel67*^*−/−*^ null mutant in hamsters. The *LdHel67*^*−/−*^ null mutant based candidate vaccine exhibited immunogenic response and a higher degree of protection against *L. donovani* in comparison to the infected control group. Further, the candidate vaccine displayed antigen-specific delayed-type hypersensitivity (DTH) as well as strong antibody response and NO production which strongly correlates to long term protection of candidate vaccine against the infection. This study confirms the potential of *LdHel67*^*−/−*^ null mutant as a safe and protective live attenuated vaccine candidate against visceral leishmaniasis.

## Introduction

Visceral Leishmaniasis (VL) is the most deadly disease among the neglected tropical diseases that targets tissue macrophages. Clinical manifestations of VL comprise high fever, hepato-splenomegaly, severe cachexia, hyper-gammaglobulinaemia and pancytopenia, which annually results in severe mortality across the globe. About 350 million people are at risk of *Leishmania* infection and annually about 500,000 new cases of visceral form of leishmaniasis occur every year. 90% cases of VL are reported in Bangladesh, Nepal, north-eastern India, north-eastern Brazil, and Sudan^1–3^. The arsenal of available drugs is not sufficient, and their prolonged utilization leads emergence of drug resistance and the toxicity^4–7^. Further unavailability of rapid diagnostic kits is also a major concern^8^. So, there is a dire need to develop a safe and cost-effective vaccine against VL. Live attenuated parasites are indispensible for eliciting protective immune response due to their persistence in the host. Development of a live attenuated vaccine requires non-virulent form of the parasite and should further study genetically modified parasites to perceive the mechanism of pathogenesis^9^. Up to now several studies using targeted gene deletion strategy have been reported for developing *Leishmania*-attenuated vaccine strains. Among the vaccine candidates studied for VL, deletion of biopterin transporter (BT1) gene in *L. donovani* reduced its infectivity in mice and this BT1deleted mutant elicited protective immunity against *L. donovani*. *L. donovani* Centrin protein lacking N-terminal domain (*NLdCen*) reduced the proliferation of the parasite in both promastigote and axenic amastigote form^10^. Later on, Centrin deleted *L. donovani* (*LdCen*^*−/−*^) also reduced the amastigotes differentiation in vitro as well as in human macrophages that induces the pro-inflammatory cytokines response in human PBMCs^11^. Moreover, *L. donovani* p27 null mutant parasite (*Ldp27*) also exhibited reduced virulence in different in vivo models and reported as nonpathogenic and renders long-term protective immunity in BALB/c mice^12^. Similarly different knockout strains like Δ*hgprt/*Δ*xprt* and Δ*aah/*Δ*hgprt/*Δ*xprt* also suppressed the parasite virulence^13,14^. *HSP70* type II deficient null mutant of *L. infantum* manifested protective efficacy in *L. major*-BALB/c mice and displayed great protection against virulent form of *L. major* promastigotes^15^. Deletion of Ufm1encoding gene in *L. donovani* malfunctioned β-oxidation of fatty acid^16^ which was reported to be regulated by Ufm1 processing peptidase activity (Ufsp) in *L. donovani.* Later on null mutant of *Ufsp* also impaired the survival of amastigotes and its pathogenesis^17^. Another null mutant of *L. donovani* lacking *Lpg2* was also unable to infect the macrophages during in vitro^18^ and in vivo condition in mouse model^19^. Similarly genetically engineered ascorbic acid-deficient live mutants of *L. donovani* also induced long lasting protective immunity against VL^20^. Recently threonyl tRNA synthetase single knockout also demonstrated attenuated infectivity of *L. donovani*^21^. DEAD-box RNA helicases represent the largest family of helicases and are widely dispersed in all three kingdoms of life. They belong to superfamily 2 (SF2) of RNA helicases which harbor an Asp-Glu-Ala-Asp (DEAD) motif that gives the name to the family^22^. *Leishmania* encodes 48–50 DEAD-box RNA helicases which have been shown to contribute in every aspect of RNA metabolism from RNA degradation, translation regulation to RNA editing^23,24^. Recently a DEAD-box RNA Helicase of 67 kDa (HEL67) has been characterized in *L. infantum* and demonstrated that it prevents ribosomal RNA degradation through an antisense rRNA-mediated pathway and translational arrest triggered by apoptotic stimuli^25^. The genomic depletion of DDX3 DEAD Box RNA Helicase (*Hel67*) significantly affects the growth of *L. infantum* promastigotes and considerably reduces amastigote differentiation and intracellular survival both in vitro and in vivo condition^26^. So far the role of DEAD Box RNA Helicase 67 has not been reported in *L. donovani*. Therefore to evaluate the role of DEAD Box RNA Helicase 67 in *L. donovani*, in this study we have constructed DEAD Box RNA Helicase 67 null mutant in *L. donovani (LdHel67*^*−/−*^) and confirmed its prophylactic efficacy as a live attenuated vaccine candidate in hamster model.

## Results

**Figure 1:**
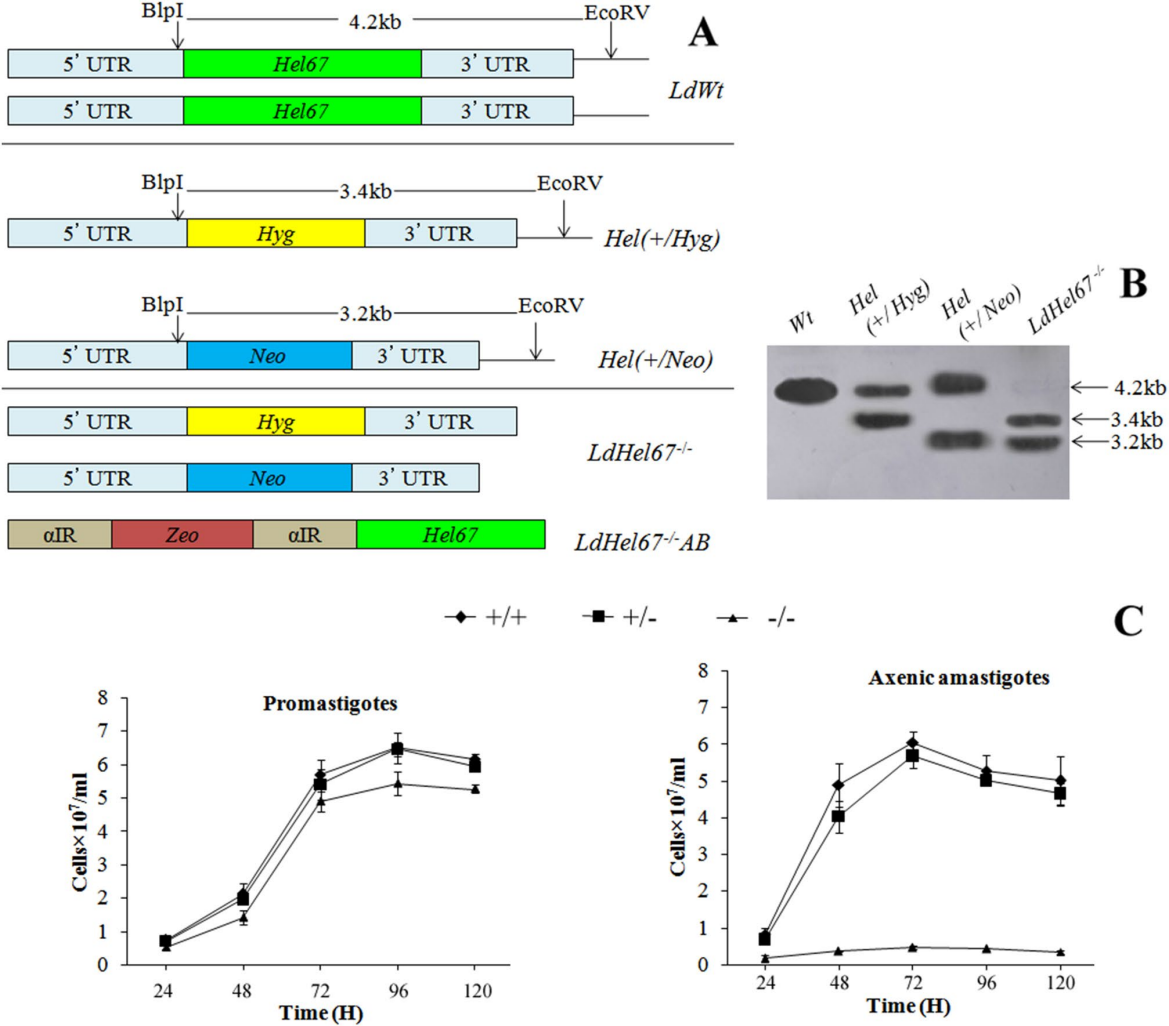
(**A**) Strategy to generate a *L. donovani* null mutant (*LdHel67*^*−/−*^) and add back/revertant mutant (*LdHel67*^*−/−*^ AB). Both alleles of the *Hel67* gene were replaced by the hygromycin phosphotransferase gene (*Hyg*) and neomycin phosphotransferase gene (*Neo*) cassettes, respectively, through homologous recombination. Add back/revertant mutant was created by expressing *Hel67* gene in pSP72αZEOα vector (**B**) Southern blot hybridization of genomic DNA digested with *BlpI* and *EcoRV* using the Hel67 3′-flank sequence as a probe. In (*LdHel67*^*−/−*^), two hybridizing bands of 3.4 kb (for the *Hyg* gene replacement) and 3.2 kb (for the *Neo* gene replacement) were detected but not the 4.2 kb *Hel67* endogenous band, (**C**) The effect of *LdHel67* disruption on the in vitro growth of *Leishmania* promastigotes and axenic amastigotes of wild type (+/+), *Hel67* single allele disrupted (+/−), and *Hel67* null mutant (−/−). The cells were grown in the absence of antibiotics. Initial cell density in the culture was 0.1 × 10^7^ cells/ml. The data represent the means ± S.D. of three independent experiments. No significant difference in growth was observed between +/− and +/+ parasites.

**Figure 2:**
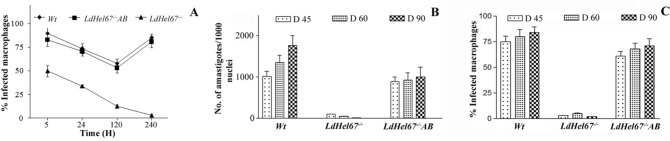
(**A**) In vitro safety efficacy of *LdHel67*^*−/−*^ null mutant in Hamster peritoneal macrophages. Infectivity of wild type (WT), *LdHel67*^*−/−*^ null mutant and *LdHel67*^*−/−*^ episomally expressing *Ld*HEL67 protein (add back/revertant; *LdHel67*^*−/−*^* AB*) parasites in hamster peritoneal macrophages at 5, 24, 120 and 240 h post infection is shown. At each time point in an experiment at least 300 total macrophages were counted. (**B**) in vivo safety efficacy of *LdHel67*^*−/−*^ null mutant in Hamster. Parasite burden in spleen of hamster infected with wild type (WT), *LdHel67*^*−/−*^ null mutant and add back/revertant *LdHel67*^*−/−*^* AB* is shown on days 45, 60, and 90 post infection. (**C**) Infectivity (% infected macrophages) of wild type (WT), *LdHel67*^*−/−*^ null mutant and *LdHel67*^*−/−*^ episomally expressing *Ld*HEL67 protein (add back/revertant; *LdHel67*^*−/−*^* AB*) parasites in hamster spleen on days 45, 60 and 90 post infection. The data presented are the mean ± S.D. of three independent experiments. Episomal expression of HEL67 protein in the *LdHel67*^*−/−*^ parasites restores positive growth in cultured macrophages (ex-vivo) as well as in hamster (in vivo).

**Figure 3:**
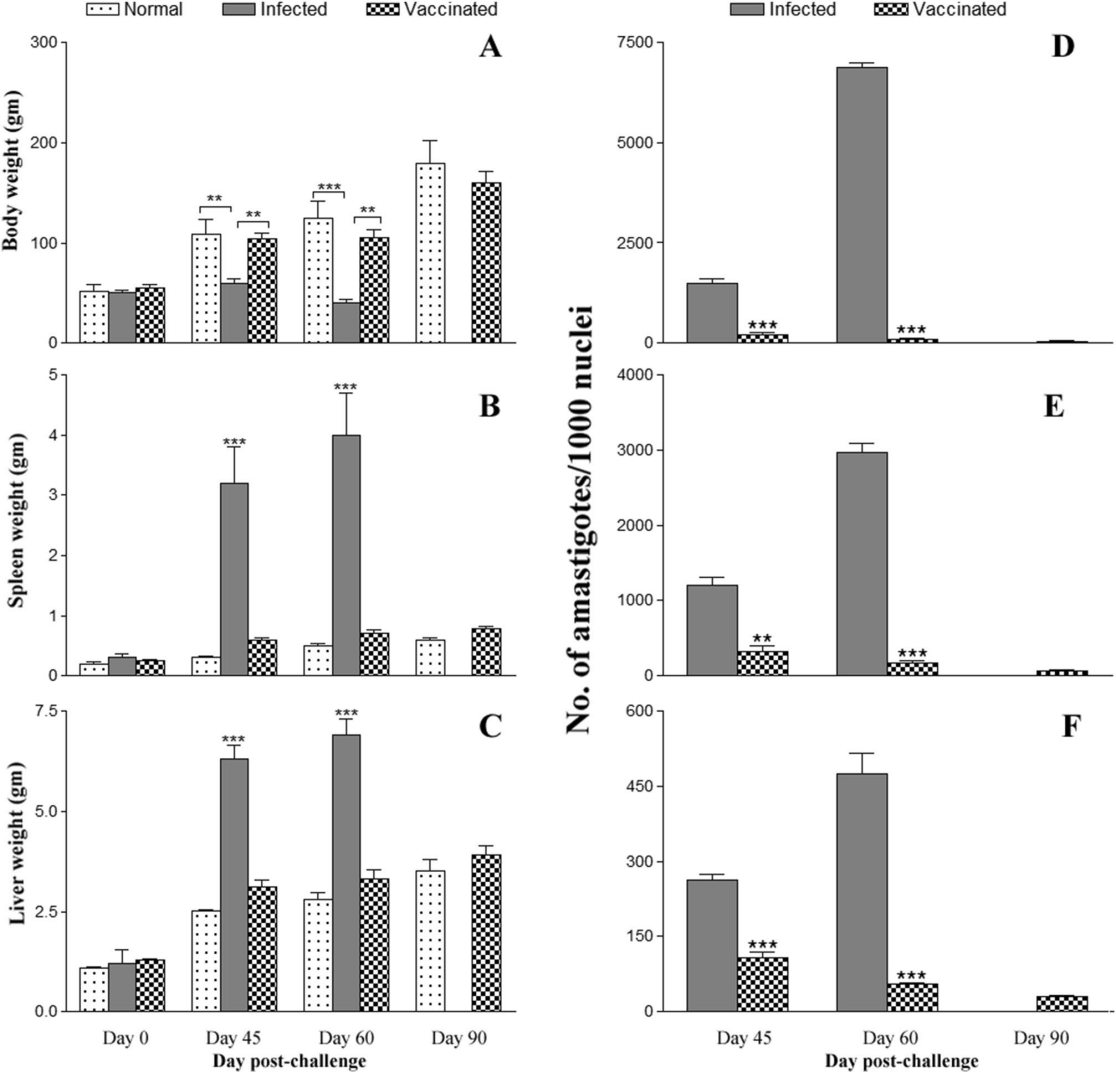
Body weight (**A**), spleen weight (**B**), and liver weight (**C**) in gm as well as parasite burden (no. of amastigotes per 1000 cell nuclei) in the spleen (**D**), liver (**E**), and bone marrow (**F**) on days 45, 60, and 90 p.c. The data for infected control group on day 90 is missing because most of the animals of infected control group were succumbed to the *L. donovani* challenge. Significance values indicate the difference between the vaccinated groups and infected group (**,*p* < 0.01; and ***, *p* < 0.001).

To investigate the role of HEL67, a *L. donovani* null mutant *LdHel67*^*−/−*^ was generated by *Hel67* gene replacement with two distinct selectable marker genes using homologous recombination strategy. The *Hyg* and *Neo* genes conferring resistance to Hygromycin B and Geneticin correspondingly were flanked at the 5′ and 3′ends with the 5′- and 3′-UTRs of the *Hel67* gene, respectively, and used in the recombination procedure (Fig. 1A). Verification of the *Hel67* gene replacement was done by PCR (Supplementary Fig. S1) and Southern blot analysis. In southern blotting analysis the absence of the endogenous band of 4.2 kb for wild-type *LdHel67* and the presence of *Hyg*- and *Neo*-hybridizing bands of 3.4 kb and 3.2 kb, respectively, authenticated deletion of both copies of *Hel67* gene (Fig. 1B) and (Supplementary Fig. S2). Removal of both the alleles of *Hel67* did not obstruct the growth of promastigote form of the null mutant (Fig. 1C left panel). Likewise, the single *Hel67* knockout (+/−) promastigotes and the axenic amastigotes also developed parallelly to the wild type control (+/+) (Fig. 1C, left panel). While the transfer of the *LdHel67*^*−/−*^ promastigotes (−/−) into the axenic amastigote specific medium, displayed a sharp decrease in the growth pattern *of LdHel67*^*−/−*^ parasite. (Fig. 1C, right panel). Further we have evaluated the ex vivo and in vivo safety efficacy of *LdHel67*^*−/−*^ null mutant. Metacyclic *LdHel67*^*−/−*^ parasites when used to infect hamster peritoneal macrophages (ex-vivo) (Fig. 2A) or when injected in susceptible golden Syrian hamsters (Fig. 2B,C) (in vivo), manifested attenuation in growth (i.e., significantly reduced number of parasites and significantly lowered the figure of % infected macrophages over time in the spleen on days 45, 60 and 90 post infection in contrast to the control). This result can be directly correlated with the deficiency of *Hel67*, because *LdHel67*^*−/−*^ AB (add back/revertant) expressing HEL67 protein episomally can revert the infectivity in hamster peritoneal macrophages (ex vivo) as well as in animal model (in vivo). Moreover, we have also evaluated the infectivity of *LdHel67*^*−/−*^ null mutant in hamsters by suppressing the immune system using cyclophosphamide (CPA) as immunosuppressant and found that *LdHel67*^*−/−*^ null mutant parasites never regained any infectivity on days 45, 60 and 90 p.i. Since, *LdHel67*^*−/−*^ null mutant could not progress any disease in hamster, so we have evaluated its prophylactic efficacy as a genetically modified live attenuated vaccine candidate. The safety efficacy of *LdHel67*^*−/−*^ null mutant was examined in hamsters on necropsy on days 45, 60 and 90 p.c. Dab smears were prepared from the spleen of experimental hamsters and parasite load was evaluated by counting the number of amastigotes/1000 cell nuclei of spleen. Immunization with *LdHel67*^*−/−*^ null mutant reduced the parasite load from spleen to a negligible amount after 45, 60 and 90 days of inoculation as compared to the hamsters infected with the wild type where the parasites were keep progressing (Fig. 3). Increase in the body weight of vaccinated hamsters similar to the normal control group clearly indicates that immunization with *LdHel67*^*−/−*^ null mutant protects hamsters from the *L. donovani* challenge. While in case of infected control group a significant loss of weight was observed (Fig. 3 A). The vaccinated group didn’t display any symptoms of splenomegaly and hepatomegaly that are commonly linked with challenge infections (Fig. 3B,C). Moreover, parasitic burden was also recorded from the spleen, liver and bone marrow and observed after staining them with the giemsa-stain. Significant decrease (*p* < 0.001) in the number of parasite was recorded from the *LdHel67*^*−/−*^ null mutant vaccinated group on days 45 and 60 and by day 90 there was almost a negligible parasites load recorded from these body parts. However, in the organs of *L. donovani* unvaccinated challenged group (infected group) significantly higher number of parasite were observed throughout the experiment (Fig. 3D–F). The data for infected control group on day 90 is missing because most of the animals of infected control group were succumbed to the *L. donovani* challenge (Supplementary Fig. S3). Further, in vitro cultivation of spleen and liver tissues from the vaccinated group of hamsters also did not show any promastigote growth even after three weeks of incubation. Lastly, hamsters vaccinated with the *LdHel67*^*−/−*^ null mutant survived against the challenge of *L. donovani* and remained healthy till the last day of experiment.

**Figure 4:**
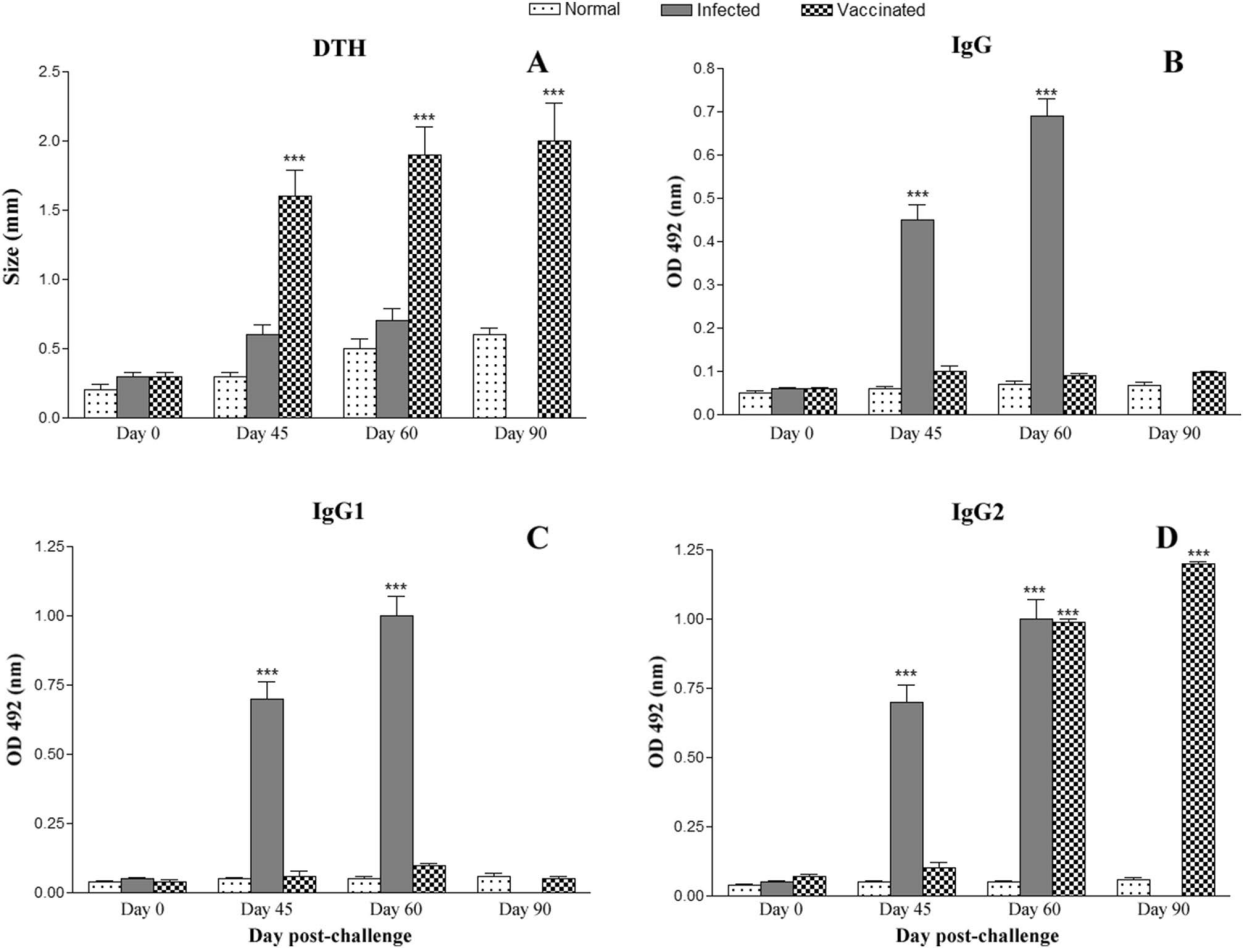
DTH response (mm) (**A**), and *Leishmania*-specific IgG (**B**) and its isotypes IgG1 (**C**) and IgG2 (**D**) in *LdHel67*^*−/−*^ null mutant vaccinated hamsters in comparison to the unimmunized infected controls, and uninfected normal hamsters on days 0, 45, 60, and 90 p.c. Significance values indicate the difference between the vaccinated groups and infected group (***,*p* < 0.001).

Immunization with *LdHel67*^*−/−*^ null mutant elicited cell mediated immunity (CMI), which was confirmed by examining the delayed type hypersensitivity (DTH) responses to specific and/or nonspecific type of antigens (Ags) in these *L. donovani* challenged hamsters at different intervals of time. *LdHel67*^*−/−*^ null mutant immunization induced hamsters to produce significant DTH responses, which increased exponentially (Fig. 4A) and elevated to the even higher level as compared to the control group (*p* < 0.001) all through the period of the experiments. Moreover estimation of serum level of leishmanial Ag-specific IgG and its isotypes (IgG1 and IgG2) from every experimental group were done by ELISA. Progression in the level of anti-*Leishmania* IgG and IgG1 were recorded from every group except the vaccinated (*LdHel67*^*−/−*^ null mutant) group; in this case level of IgG and IgG1 remained as background levels of the infected and normal control group (Fig. 4B,C). While *LdHel67*^*−/−*^ null mutant immunized group was the only group that displayed one–twofold increase in the concentration of IgG2 as compared to others (*p* < 0.001) (Fig. 4D). Thus a consistent elevation in the IgG2 level manifested effective immune responses that may suggest boosting the cell-mediated immunity.

**Figure 5:**
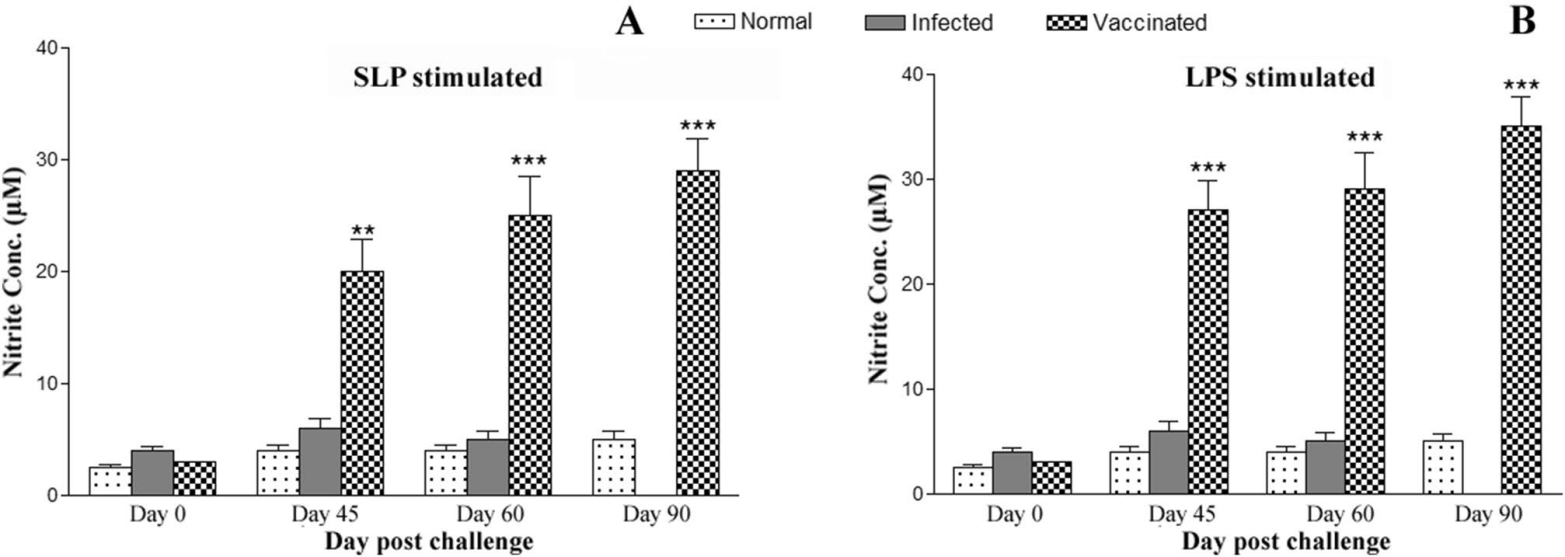
NO production (μM) to SLP (**A**) and LPS (**B**) in the naive macrophages co-incubated with supernatants of lymphocytes isolated from *LdHel67*^*−/−*^ null mutant-immunized hamsters in comparison to the unimmunized infected controls and uninfected normal hamsters on days 0, 45, 60, and 90 p.c. Significance values indicate the difference between the vaccinated groups and infected group (**,*p* < 0.01; and ***, *p* < 0.001).

A significant difference between the control and experimental groups of hamsters was observed to produce NO for leishmanicidal activity by Lymphocyte-mediated activation of macrophages. Incubation of naive macrophages with the supernatant from stimulated lymphocytes of *LdHel67*^*−/−*^ null mutant vaccinated hamsters induced the production of nitrite to a significant (*p* < 0.001) level which was three–fourfold higher over the infected control group on day 45. Later on the nitrite concentration was increased on days 60 and 90 p.c. In contrast, the unvaccinated normal and infected groups produced relatively a very low level of nitrite on day 45 (Fig. 5A). In the same way, LPS (100 μg/ml, used as positive control)-stimulated cells from the immunized group also demonstrated good and significant (*p* < 0.001) amount of nitrite until day 90 p.c, whereas, a low level of nitrite production was displayed by infected control group till they died (Fig. 5B).

## Discussion

The present report describes for the first time a gene knockout for *Hel67* in *L. donovani* and the importance of such a gene for amastigote differentiation and infectivity in the *Leishmania* parasite. Previous studies demonstrated that major role of DDX3 DEAD Box RNA Helicase (*Hel67*) in mitochondrial protein quality control under normal growth conditions and particularly upon stress by preventing ROS-mediated damage in *L. infantum*^26^. In our study, *LdHel67*^*−/−*^ null mutants are also unable to grow as amastigote form. So we can speculate that they may be unable to cope up with the ROS-mediated damage under the stress condition. We think that the specific attenuation in the genetic makeup prevents the parasite from reverting back to its virulent form, a concern typically arising for attenuated organisms that are generated by random genomic mutations. Deletion of *Hel67* specifically weakened the amastigote form of the parasite that replicates inside macrophages, while on the other hand there was no obstruction on the growth of the promastigote form. Further, we have assessed the infectivity of *LdHel67*^*−/−*^ null mutant in hamster peritoneal macrophages (ex-vivo) as well as in hamsters (in-vivo). Remarkably we have observed a significant reduction in the number of infected macrophages (ex-vivo) as well as intracellular amstigotes (in-vivo) in the spleen at different time intervals as compared to the infected control group. Such inability of the *LdHel67*^*−/−*^ null mutant parasite to grow inside the macrophages milieu signals deficiency in parasite virulence. This reduction in virulence is the direct consequence of *Hel67* deficiency because *LdHel67*^*−/−*^ null mutant cells expressing HEL67 protein (add back/revertant) from a transfected plasmid were rescued for growth both in macrophages and in the hamster model. Due to these phenotypic changes particularly the lack of virulence of *LdHel67*^*−/−*^ null mutants we have anticipated to evaluate this null mutant as a genetically modified live attenuated vaccine candidate against experimental VL. Since *LdHel67*^*−/−*^ null mutants were able to grow only as a promastigote stage, so it is quite possible to grow parasites for vaccine trials in bulk amount. To evaluate the prophylactic efficacy of *LdHel67*^*−/−*^ null mutant it was further tested as a genetically modified live attenuated vaccine candidate in Syrian golden hamsters. All the *LdHel67*^*−/−*^ vaccinated hamsters challenged with the infection of *L. donovani* continued to survive and stayed healthy even after 180 days of infection. While the unvaccinated infected animals could not survive more than 2–3 months p.c. Further, an increase in body weight, decrease in the weight and sizes of visceral organs, and reduction of parasite burden indicating that vaccination with *LdHel67*^*−/−*^ null mutant induced optimum prophylactic efficacy. The immune mechanism(s) answerable for defense in this vaccine model or any other leishmanial vaccine model has not been yet well-defined. However, a key element that is thought to be involved in healing from leishmaniasis is the development of strong cell-mediated immunological (CMI) responses like Delayed type hypersensitivity (DTH) responses and nitric oxide (NO production)^27–29^. The occurrence of parasite-specific DTH response at a low level in the infected control group indicates the progression of disease in hamsters. Whereas the *LdHel67*^*−/−*^ null mutant vaccinated group of hamsters elicited the strongest DTH response suggesting a correlation between the cell-mediated immune responses and immunity to infection in this model. In the same way, an increase in the NO level was also recorded from the cells of vaccinated hamsters stimulated with SLP. This increase in the production of NO indicates a defense mechanism of activated macrophages to stop the parasite growth inside the host macrophages. Besides the cell-mediated immunity, VL is also concerned with the production of an increased level of *Leishmania*-specific immunoglobulin. It has already been reported that the titer of IgG and IgG1 Abs increase during *L. donovani* infection^30^. While the absence of these Abs in the vaccinated group can be correlated with decreased parasite load in the vaccinated group. However, a significant increase in the IgG2 levels only in vaccinated animals is indicative of enhanced CMI.

In summary, the present study demonstrates a gene knockout of *Hel67* in *L. donovani* and its importance for growth and differentiation of the *Leishmania* parasite. Further a noticeably high immunogenicity and protective efficacy of the live attenuated *LdHel67*^*−/−*^ against *L. donovani* infection was recorded. The candidate vaccine raised a strong *Leishmania* specific IgG2 response and NO production that lead to enhance the CMI. Since all the vaccinated hamsters remained healthy and survived till the end of the experiment, this strongly correlates to a long lasting protection of the candidate vaccine against the infection. Further, immunological parameters such as cytokine profiling and lymphoproliferative assay in hamster and other animal models are required to investigate in detail. Moreover, inactivation of the *Hel67*gene in the amastigote form of other heterologous *Leishmania* species can also be explored. Further the similar strategy could also be utilized for the development of live attenuated vaccine candidate against various other tropical diseases like malaria and trypanosomiasis.

## Materials and methods

### Parasite culture and targeted gene deletion

*Leishmania donovani* WHO reference strain Dd8 was employed in this study and cultured in vitro as described elsewhere^31^. Promastigote were cultured in RPMI-1640 medium (pH 7.3) supplemented with 10% heat-inactivated FCS 1% penicillin (100 U/ml) and streptomycin (100 mg/ml) at 25 °C and as axenic amastigotes in MAA-20 medium supplemented with 20% FCS and grown at pH 5.5 and 37 °C in a 5% CO2 atmosphere. To inactivate the DEAD Box RNA helicase (*Hel67*) gene of *L. donovani* (LdBPK_320410.1) (TriTrypDB; https://tritrypdb.org) with hygromycin and neomycin phosphotransferase (*Hyg* & *Neo*) genes, Nine set of primers were designed and named as Primer A, HYG-B, NEO-B, HYG-C, NEO-C, HYG-D, NEO-D, E and F (Supplementary Fig. S4 upper panel) (Table 1). Primers A and B were used to amplify 5′ flanking region using genomic DNA, HYG-C/NEO-C and HYG-D/NEO-D were used to amplify hygromycin and neomycin phosphotransferase genes using specific plasmids and primer E and F were used for amplification of 3′ flanking region from genomic DNA of the *L. donovani*. To construct add back mutant (*LdHel67*^*−/−*^* AB*) *LdHel67* ORF was amplified (using ORF primers, Table 1) by PCR (Bio-Rad, India) and cloned into the *XbaI* and *HindIII* sites of vector pSP72αZEOα expressing the zeomycin (ZEO) marker^25^. The Plasmids psp72-αNEOα, psp72Y-HYGα and pSP72αZEOα were procured from Prof. Barbara Papadopoulou, Laval University, Quebec, Canada. Genomic DNA was isolated using DNAzol (Invitrogen, Thermo Fisher Scientific) as per manufacture’s protocol. HYG and NEO constructs containing 5′ and 3′ flanking regions of DEAD Box RNA Helicase of *L. donovani* were cloned in pGEM-T Easy vector (Promega,USA) (T/A cloning) by blue white colony screening. After successful screening appropriate clone of HYG and NEO constructs were sequenced before transfecting. The NEO and HYG constructs were released from pGEMT Easy vector using *NotI* enzyme. Both HYG and NEO constructs were transfected in *Leishmania* promastigotes using electroporator (Gene Pulser Xcell electroporation system, Bio-Rad, India). as described^32^. Stable transfectants were selected and cultivated with either 0.025 mg/ml G-418 (Sigma-Aldrich) or 0.080 mg/ml Hygromycin-B (Sigma-Aldrich) or in both. Transfectants were finally plated on culture plate containing 80 μg/ml of hygromycin B or 25 mg/ml G418. Transfectants isolated from the plates were subsequently expanded in liquid medium containing 40 μg/ml of hygromycin B or 12.5 mg/ml G418^25^. Genomic DNA isolated from these transfectants was used in Southern blot/ PCR analyses to confirm the loss of one allele of *Hel67*. Such a cell line in which one allele of the *LdHel67* gene was substituted by the *Hyg* and *Neo* gene was subjected to the next round of transfection using construct 2, and transfectants were selected on culture containing 80 μg/ml of hygromycin B and 40 μg/ml of G418 antibiotics. The transfectants were analyzed for the complete knockout of *Hel67* gene and the presence of both *Hyg* and *Neo* genes using Southern blot hybridization^33^. Southern blot hybridization was performed following standard procedures. Biotin labelled probe from PCR fragment using primers E and F was synthesized using Biotin Decalabel DNA Labeling kit (Thermo Scientific, USA) (Supplementary Fig. S4 lower panel) and was detected by Biotin Chromogenic Detection kit (Thermo Scientific, USA) as per the manufacturer’s protocol. Single or both alleles deleted parasites were grown both as promastigotes and axenic amastigotes in the absence of antibiotics except the single knockout (+/−) that was grown in the presence of hygromycin B or G-418 to avoid elimination of the *Hyg* or *Neo* genes by the duplication of the single *Hel67* allele^34^.

**Table 1:** Primers used to inactivate and to over express the DEAD Box RNA Helicase 67 (*Hel67*) gene of *L. donovani.*

Primer	Sequence
Primer A (forward)	5′-AGTATAGCAGGGATGGAGG-3’
HYG-B (reverse)	5′-GGTGAGTTCAGGCTTTTTCATGATTCCTGCTTAGCAAACG-3’
NEO-B (reverse)	5′-CAATCCATCTTGTTCAATCATGATTCCTGCTTAGCAAACG-3’
HYG-C (forward)	5′-ATGAAAAAGCCTGAACTCACC-3’
NEO-C (forward)	5′-ATGATTGAACAAGATGGATTG-3’
HYG-D (reverse)	5′-CATTTCTCTCGCCTGCGAACCTATTCCTTTGCCCTCGGACGAG-3’
NEO-D (reverse)	5′-CATTTCTCTCGCCTGCGAACTCAGAAGAACTCGTCAAGAAG-3’
Primer E (forward)	5′-ATGGAGTAGTAAAACTCCGTGT-3’
Primer F (reverse)	5′-GACAGAGAAAAGCGTGTGTG-3’
*Ld*HEL67 ORF forward	5′-GCTCTAGAATGTATAAGAATCAGGCGCAAC-3’
*Ld*HEL67 ORF reverse	5′-CCAAGCTTCTACTGACCAAAGACGTCAGATCG-3’

### Animals

Laboratory inbred female golden hamsters (*Mesocricetus auratus*, 45–50 g) were purchased from the Central Drug Research Institute (CDRI-CSIR), Lucknow, India and were used for experimental purposes with prior approval of the animal ethics committee (Protocol No. KUDOPS/109) of Kumaun University, Nainital (Uttarakhand). All methods were carried out in accordance with relevant guidelines and regulations.

For isolation of peritoneal macrophages, peritoneal cavity of hamsters was treated with Thioglycollate for allowing inflammatory response to proceed for 4 days, and then euthanize. Isolated peritoneal macrophages were then resuspended at 1.8 × 10^5^ cells/ml in RPMI medium plated in 0.5 ml on six chamber tissue culture slides (Nunc Lab-Tek) and incubated for 8 days for differentiation into macrophages^6^. The differentiated macrophages were infected with stationary phase cultures of promastigotes (10:1, parasite to macrophage ratio). After incubation for 5 h at 37 °C in 5% CO_2_, the free extracellular parasites were removed by repeated washings in RPMI, and the cultures were incubated in macrophage culture medium for maximum of 240 h. At 5, 24, 48, 120, and 240 h post-infection, the culture medium was removed from a sample of the culture slides, and the slides were air-dried, fixed by immersion in absolute methanol for 5 min at room temperature, and stained using geimsa stain^10^. For each culture, a minimum of 300 macrophages were counted. The results are expressed either as percentages of macrophages that were infected by *Leishmania* or as the mean number of parasites/infected macrophage^10^. Prior immunization, amsters were injected intracardially with 10^7^ cells of *LdHel67*^*−/−*^ null mutants and *L. donovani* WT virulent strain to check the safety efficacy. Days 45, 60 and 90 post infection hamsters were sacrificed to check the parasite burden in Spleen. Furthermore before evaluating the immunization study, infectivity of *LdHel67*^*−/−*^ null mutant parasites was also checked by immuno-suppression. For that hamsters were administered intraperitoneally with a weekly dose of 150 mg/kg cyclophosphamide (CPA) from days 0 to 21 p.i. and the infectivity was evaluated on days 45, 60 and 90 p.i.

For in vivo experimental study, a total of 60 Syrian golden hamsters (40–45 g) were divided into three groups with 15–25 animals in each (Supplementary Fig. S5). The group 1 and 2 served as controls and group 3 as the main experimental group: group 1, unvaccinated and unchallenged (normal control); group 2, unvaccinated and challenged (infected control); group 3, *LdHel67*^*−/−*^ null mutant vaccinated (vaccinated group). The schedules of the experiment for vaccination/immunization were conducted as described below^31^:

**Day 0 vaccination**: Hamsters of group 3 were injected intramuscularly (i.m.) in the thigh muscle of the hind leg with 10^5^ *LdHel67*^*−/−*^ null mutant parasites.

**Day 21 after vaccination**: Each animal of groups 2 and 3 was challenged intracardially with 10^7^ late log-phase promastigotes of the Dd8 strain of *L. donovani*. On days 0, 45, 60 and 90 post challenge (p.c.), 3–5 hamsters from each group were euthanized for parasitological and immunological estimation of VL progression.

### Measurement of delayed-type hypersensitivity (DTH) in hamsters

DTH was performed through intra-dermal injection of 50 μg/50 μl of Soluble *Leishmania* Protein (SLP) in PBS into one footpad and PBS alone into the other one of each of the vaccinated and unvaccinated controls. The response was accessed 48 h of post inoculation by measuring the difference in footpad swelling between the two with and without SLP for each animal^31,35^.

### Assessment of parasitic burden in vaccinated hamsters

The prophylactic efficacy of all of the experimental groups was evaluated on necropsy at different time period, i.e., on days 0, 45, 60, and 90 p.c. The dab smears or touch blots from the spleen, liver and bone marrow (femur bone) of experimental animals were prepared and assessed by counting the number of amastigotes/1000 cell nuclei in each organ^31^.

### Immunological assays

For evaluation of cellular and Ab responses, peritoneal exudate cells, inguinal lymph nodes, and blood were collected from hamsters on necropsy at various time intervals, i.e., day 0, 45, 60, and 90 p.c ^31^.

### Assessment of level of NO activity in macrophages of vaccinated hamsters

The presence of nitrite (NO_2_^-^) content was assessed using Griess reagent in the culture supernatants of naive hamster peritoneal macrophages after the exposure with supernatant of stimulated lymphocyte cultures. Briefly, isolated peritoneal macrophages were suspended in culture medium and plated at 10^6^ cells/well and exposed to the supernatants of the above described 5-day-old Ag-stimulated lymphocyte cultures from all of the study groups. The supernatants (100 μl) collected from macrophage cultures 24 h after incubation were mixed with an equal volume of Griess reagent (Sigma-Aldrich) and left for 10 min at room temperature. The absorbance of the reaction was measured at 540 nm in an ELISA reader (BioTek, USA)^36^.

### Determination of antileishmanial antibody responses in hamsters

The levels of antileishmanial Abs-IgG and its isotypes, IgG1 and IgG2, in sera samples from hamsters of different groups were measured as described earlier^37^. The 96-well ELISA plates (Nunc Lab-Tek) were coated with SLP (0.2 μg/100 μl/well) overnight at 4 °C and blocked with 1% BSA at room temperature for 1 h. The optimum dilution of sera was standardized at 1/200 for IgG, IgG1, and IgG2 for 2 h at room temperature. HRP-conjugated goat anti-hamster IgG (H + L) and biotin-conjugated mouse anti- Armenian and anti-Syrian hamster IgG1 (for IgG1) as well as mouse anti-Syrian hamster IgG2 were added for 1 h at room temperature at 1/800 dilutions. IgG1 and IgG2 plates were further incubated with streptavidin-conjugated peroxidase (Sigma-Aldrich) for 1 h. Finally, the substrate O-phenylenediaminedihydrochloride (OPD) was added and the plate was read at 492 nm^38^.

### Post-challenge survival

Survival of hamsters belonging to vaccinated group (group 3) was checked up to 6 month p.c. in comparison to the normal hamsters (group 1)^31^. Animals in all of the groups were given proper care and were observed for their physical conditions until their survival period. Survivals of individual hamsters were recorded and mean survival period was calculated.

### Statistical analysis

Results are expressed as mean ± S.D. of three to five individual animals per group at designated time points. Three replicates were done. The results (pooled data of three experiments) were analyzed by one-way ANOVA followed by Tukey’s post test or by unpaired t-test. All of the analyses were done using GraphPad Prism (version 5) software.

## Supplementary information

Supplementary Figures.
