# Supplementary material for: DDX3 DEAD-box RNA helicase (Hel67) gene disruption impairs infectivity of Leishmania donovani and induces protective immunity against visceral leishmaniasis
Source: Sci Rep. 2020 Oct 26;10:18218. doi: 10.1038/s41598-020-75420-y (PMC7589518; doi:10.1038/s41598-020-75420-y)
Supplement: Supplementary file 1 — Supplementary Figures. [file 41598_2020_75420_MOESM1_ESM.pdf]

Supplementary data

DDX3 DEAD-Box RNA Helicase (*Hel67*) gene disruption impairs infectivity of *Leishmania donovani* and induces protective immunity against Visceral Leishmaniasis

Satish Chandra Pandey[1,2], Veena Pande[2], Mukesh Samant[1*]

[1]Cell and Molecular biology laboratory, Department of Zoology, Kumaun University, SSJ Campus, Almora (Uttarakhand), India

[2]Department of Biotechnology, Kumaun University, Bhimtal Campus, Nainital, (Uttarakhand), India

[*] **Corresponding Author**

Mukesh Samant, Ph.D.

Assistant Professor

Cell and Molecular biology laboratory

Department of Zoology, Kumaun University,

SSJ Campus, Almora (Uttarakhand), INDIA

E-mail: mukeshsamant@gmail.com

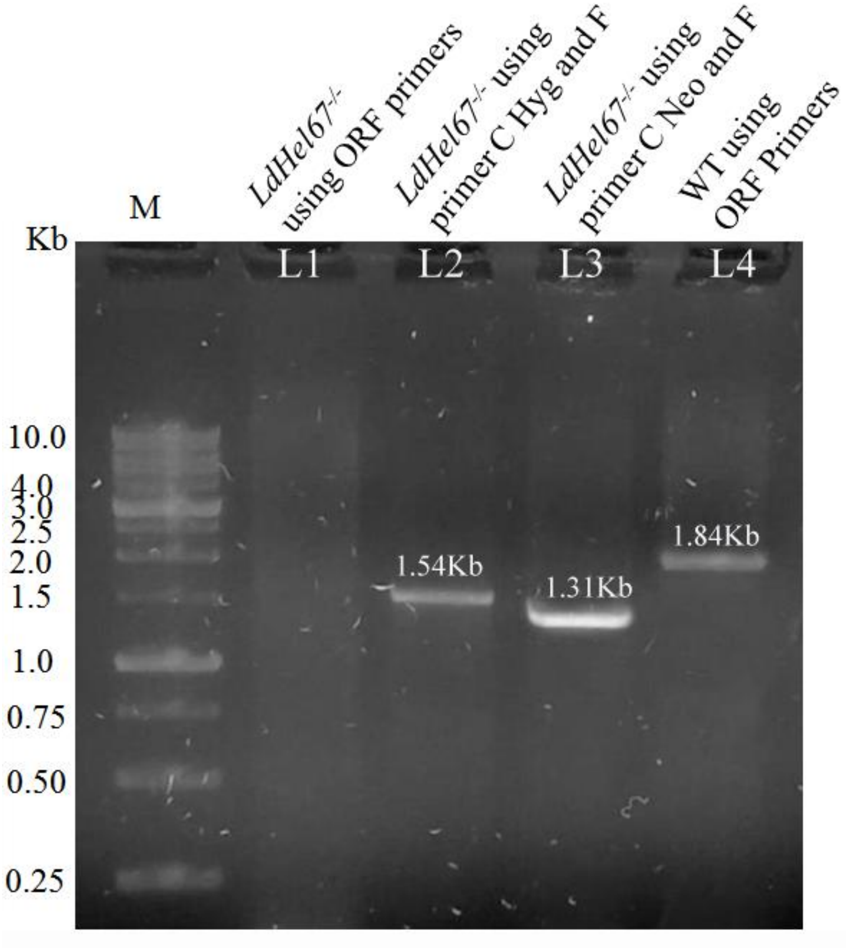

Supplimentary figure S1- PCR analysis to confirm the integration of *Hyg* and *Neo* genes. M-molecular marker; Lane 1- No PCR amplification observed for *LdHel67*$^{-/-}$ null mutant using forward and reverse *Ld*HEL67 ORF primers; Lane 2- PCR amplified bands located at 1.54Kb for *LdHel67*$^{-/-}$ null mutant using forward primer C Hyg and reverse primer F; Lane 3- PCR amplified bands located at 1.31Kb for *LdHel67*$^{-/-}$ null mutant using forward primer C Neo and reverse primer F; Lane 4- PCR amplified bands located at 1.84Kb for wild type control using forward and reverse *Ld*HEL67 ORF primers.

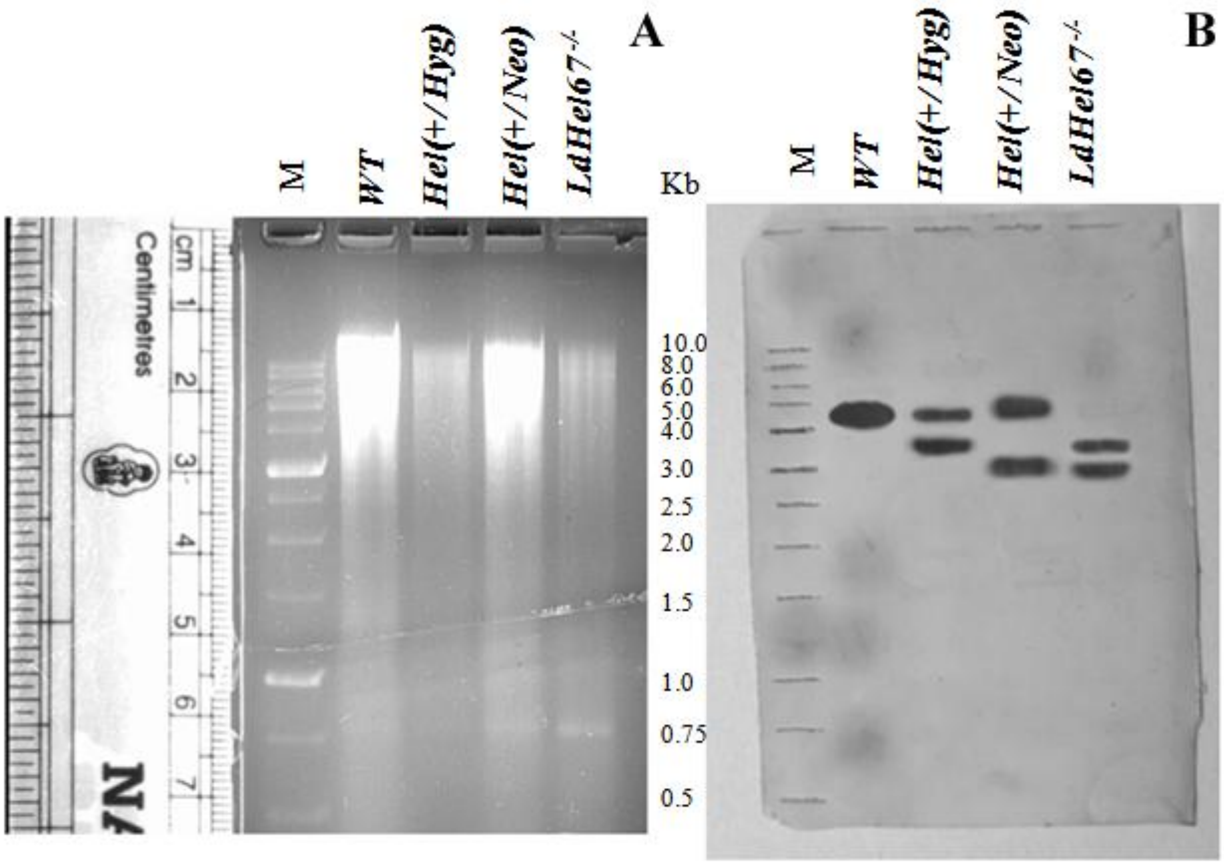

Supplementary figure S2- (A) Genomic DNA of WT, Hel (+/Hyg), Hel (+/Neo) and *Ld*Hel67$^{-/-}$ digested with *BlpI* and *EcoRV* was resolved on 1% agarose gel electrophoresis (panel A) and transferred to nylon membrane, (B) The membrane was probed using biotin labeled 3' flank sequence. Desired fragments were detected using Biotin Chromogenic Detection kit (panel B). Size of the molecular weight marker on the membrane is based on the position of the ruler on the gel in panel A.

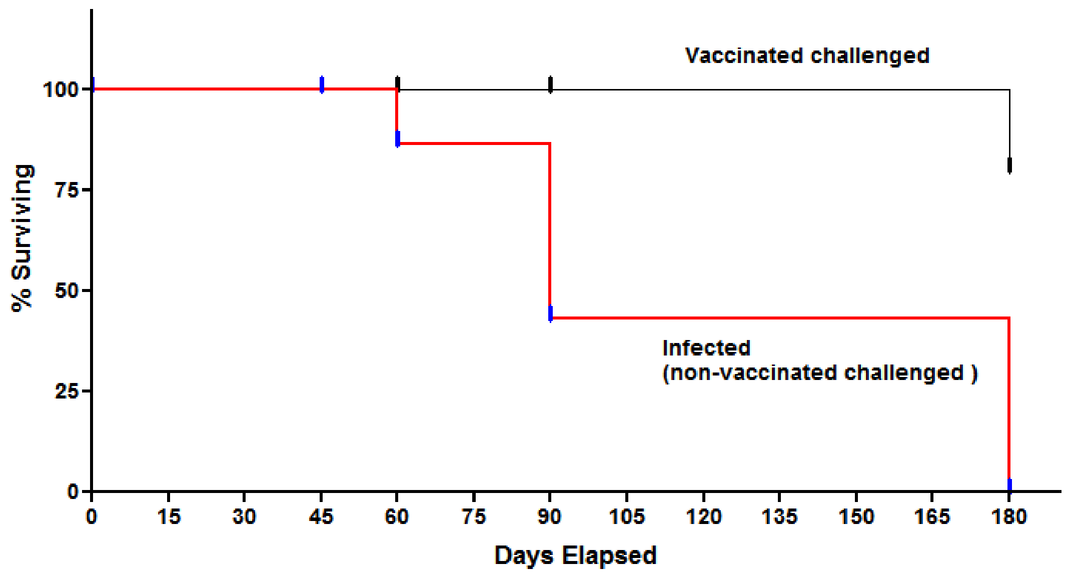

Supplementary figure S3- Kaplan-Meier mortality curve for the % survival of infected (non-vaccinated challenged) versus vaccinated challenged hamsters. Five animals were sacrificed from both groups on different time points (days 45, 60, 90). Till day 60 p.c. two animals and till day 90 p.c. all animals were succumbed to the *L. donovani* challenge in infected (non-vaccinated challenged) group. However almost all animals were survived till day 180 p.c.

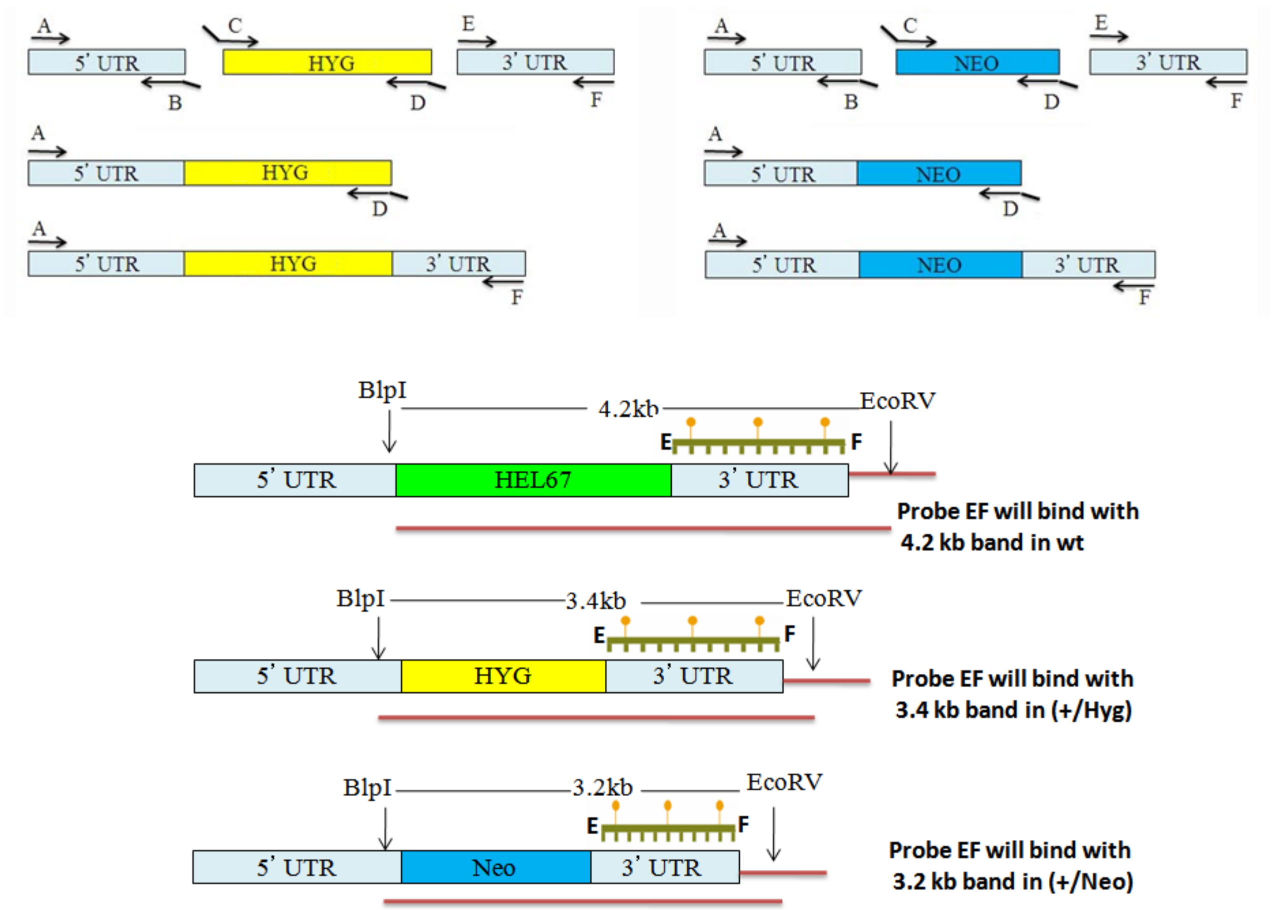

Supplementary figure S4- Strategy used for the generation of AF Hyg and AF Neo constructs using different sets of primers and southern blotting. Upper panel showing the primer binding sites in genomic DNA as well as in plasmids (psp72Y-HYGα and psp72-αNEOα) containing Hyg and Neo resistant genes. Lower panel showing binding of EF probe with BlpI and EcoRV digested fragments of wild type as well as (+/Hyg) and (+/Neo) mutants.

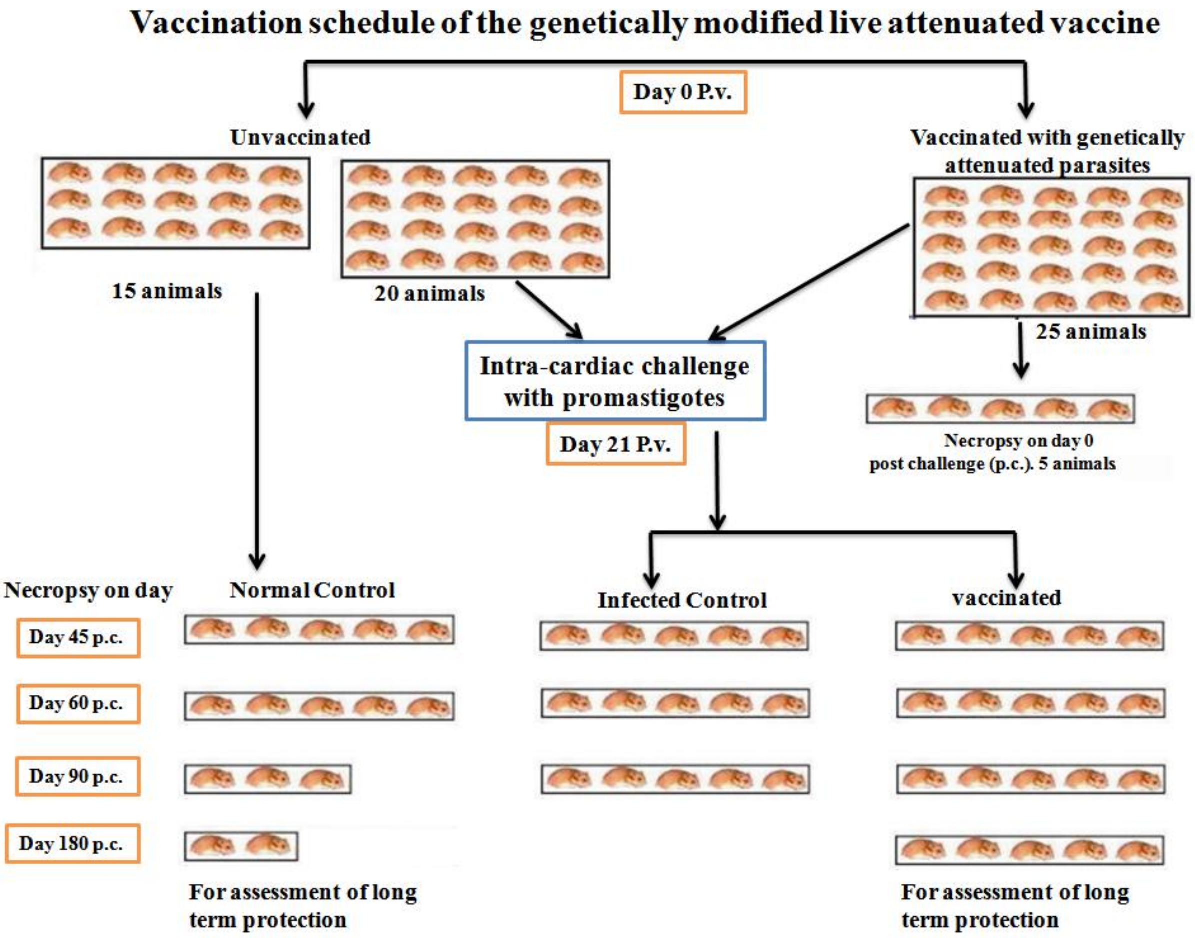

Supplementary figure S5- Schematic representation of genetically modified live attenuated vaccination schedule in hamsters.

Group [1] -Unvaccinated and unchallenged (Normal control).

Group [2] -Unvaccinated and challenged (infected control).

Group [3]- *LdHel67*$^{-/-}$ null mutant vaccinated and challenged (vaccinated group).
